# A rapid and low-cost estimation of bacteria counts in solution using fluorescence spectroscopy

**DOI:** 10.1007/s00216-017-0347-1

**Published:** 2017-04-07

**Authors:** Rachel Guo, Cushla McGoverin, Simon Swift, Frederique Vanholsbeeck

**Affiliations:** 1 The Dodd-Walls Centre for Photonic and Quantum Technologies, Department of Physics, The University of Auckland, Private Bag 92019, Auckland, 1142 New Zealand; 2 Department of Molecular Medicine and Pathology, The University of Auckland, Private Bag 92019, Auckland, 1142 New Zealand

**Keywords:** Bacteria, Fluorescence spectroscopy, Acridine orange, Enumeration, Microbial contamination

## Abstract

The fluorescence spectrum of bacterially bound acridine orange (AO) was investigated to evaluate its use for the rapid enumeration of bacteria. *Escherichia coli* ATCC 25922 samples were stained with 2 × 10^−2^, 2 × 10^−3^ or 2 × 10^−4^% *w*/*v* AO, followed by 3, 2 or 0 washing cycles, respectively, and fluorescence spectra were recorded using a fibre-based spectroscopic system. Independent component analysis was used to analyse the spectral datasets for each staining method. Bacterial concentration order of magnitude classification models were calculated using independent component weights. The relationship between fluorescence intensity of bound AO and bacterial concentration was not linear. However, the spectral signals collected for AO stain concentration-bacterial concentration pairs were reproducible and unique enough to enable classification of samples. When above 10^5^ CFU ml^−1^, it was possible to rapidly determine what the order of magnitude of bacterial concentration of a sample was using a combination of two of the sample preparation methods. A relatively inexpensive (around US$10 per test) rapid method (within 25 min of sampling) for enumeration of bacteria by order of magnitude will reduce the time and cost of microbiological tests requiring gross concentration information.

**Electronic supplementary material:**

The online version of this article (doi:10.1007/s00216-017-0347-1) contains supplementary material, which is available to authorized users.

**Graphical Abstract:**
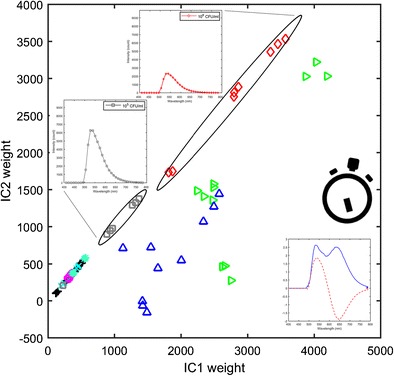
Fluorescence spectra of bacterially bound acridine orange (AO) were used for the rapid enumeration of bacteria. Order of magnitude bacterial concentration classification models were calculated using independent components analysis of these fluorescence spectra. When above 10^5^ CFU ml^−1^, it was possible to rapidly determine the order of magnitude of bacterial concentration of a sample using a combination of two sample preparation methods

## Introduction

The enumeration of microorganisms, and especially bacteria, is a core task for many microbiologists. The information obtained may be used to count contaminants, e.g. to evaluate the safety of food products and water [1], to count survivors, e.g. to evaluate the effectiveness of antimicrobial agents and processes [2], and to determine the viability of inocula for applied biological processes, e.g. for bioremediation or fermentations [3].

Agar plate counts are routinely used for bacterial enumeration; a simple technique requiring serial dilution of samples to ensure the growth of isolated colonies from single cells, and incubation for an appropriate amount of time (overnight or longer) for colonies to become visible. Consequently, this method is labour intensive, will only detect cultivable cells and is slow to return results. To overcome these limitations, culture-independent techniques have been developed, but these often increase costs with the need for more skilled staff, expensive equipment and/or consumables, sometimes with a substantial increase in the limit of detection [4, 5].

In pursuit of rapid, cultivation-independent methods to quantify bacteria, fluorescence detection has proven promising. Acridine orange (AO) is one of the most commonly used fluorescence dyes for cell enumeration and has been for the last three decades [6–8]. AO is an inexpensive nucleic acid stain, fluorescing green when it binds to double-stranded DNA and red orange when it binds to single-stranded DNA or RNA. The most popular method known to reliably detect AO-stained bacteria is fluorescence microscopy, as used in the AO direct count (AODC) method (final AO concentration 1 × 10^−2^% *w*/*v*) developed for aquatic samples [9, 10] and the direct epifluorescence filter technique method (final AO concentration 2.5 × 10^−2^% *w*/*v*) developed for milk and food samples [11, 12].

The staining of bacteria with fluorescence dyes is effective for enumeration; however, microscopy is laborious and requires automation for an efficient process. The use of fluorescence dyes, including AO, propidium iodide, ethidium bromide and SYTO BC, with flow cytometry has been successfully automated in the BactoScan system, which allows for the routine assessment of milk for bacterial counts [13, 14]. The detection limits of fluorescence microscopy and flow cytometry have been reported to be in the order of 10^3^–10^4^ CFU ml^−1^ [11, 15, 16]. However, these techniques require expensive equipment.

In this study, we used a portable and cost-effective fluorimeter to enumerate AO-stained bacteria. We have used independent component analysis (ICA) to analyse and interpret the fluorescent spectra produced. The data acquired has been used to develop a classification model for determining the order of magnitude of bacterial count from the AO-bacteria fluorescence spectra collected to enumerate bacteria in an unknown sample.

## Materials and methods

### Bacterial strain and growth condition

*Escherichia coli* American Type Culture Collection (ATCC) 25922 was obtained from the ATCC through Cryosite Ltd. (Granville, NSW, Australia). *E. coli* was cultured overnight in Difco tryptic soy broth (TSB) (Fort Richard, Auckland, New Zealand) and subsequently sub-cultured in fresh TSB (20× dilution) and grown to reach an optical density of 0.5 at 600 nm (1 cm path length) to give a suspension of the order of 10^8^ CFU ml^−1^. All broth cultures were grown at 37 °C and aerated with orbital shaking at 200 rpm.

### Samples

The calibration data (*N* = 104 samples and *n* = 275 spectra, on average three per sample) for each staining protocol was comprised of measurements from at least three samples per order of magnitude of bacterial concentration. The data were collected over a total of 13 days with fresh cultures for each day of experiment and each replicate made on a different day. Each sample was treated with only one staining protocol. Six serial tenfold dilutions of a subculture were prepared in sterile deionised water to yield a series of bacterial suspensions from 10^2^ to 10^8^ CFU ml^−1^. Calibration samples were prepared using these preparations. Validation samples (*N* = 13, each stained with the three staining protocols considered in this paper) were measured in 1 day and included a free dye sample and suspensions of bacterial concentrations in the range of 10^4^–10^8^ CFU ml^−1^.

### Standard plate count

Bacterial number in the samples was determined using the standard plate count method. For each dilution series, the sample assumed to have 10^2^ CFU ml^−1^ was used to inoculate Difco tryptic soy agar (TSA) plates (Fort Richard, Auckland, New Zealand). This assumption was based on the optical density of the most concentrated bacterial solution. For each plate count, a 100-μl aliquot of the sample was spread evenly onto a TSA plate and incubated at 37 °C overnight. For each tested sample, duplicate plate counts were made.

### Staining procedures with acridine orange

Stock 2% *w*/*v* AO in water was obtained from Sigma-Aldrich Corporation (Sydney, Australia) and diluted 10,100 and 1000 times in distilled water to give working solutions with final concentrations of 0.2, 0.02 and 0.002% *w*/*v* of AO. All these staining concentrations were tested with different numbers of washing cycles to determine how free AO can be efficiently removed from the sample. Only three staining/washing combinations yield spectra with significant differences per bacteria concentrations as determined from a principal component analysis of collected spectral data. These three staining protocols were investigated:

1. Final stain concentration 2 × 10^−2^% AO followed by three washing cycles,
2. Final stain concentration 2 × 10^−3^% AO followed by two washing cycles and
3. Final staining concentration 2 × 10^−4^% AO with no washing.

Samples were prepared in amber or aluminium foil-covered microcentrifuge tubes. Each sample consisted of a 900-μL aliquot of bacteria suspension or distilled water for free dye samples, which was incubated with 100 μL of working AO solution. Amber or aluminium foil-covered microcentrifuge tubes were used to limit exposure to external light sources and subsequent photodegradation. Samples were mixed for 3 min by vortexing and then rested in the dark for 15 min. Samples were then subjected to washing, as required, to reduce the concentration of unbound dye in the sample. A single washing cycle consisted of several consecutive steps: centrifuging the sample at 4300×*g* for 5 min at room temperature, removing 970 μL of supernatant, adding 970 μL of distilled water and finally vortexing the sample for 2 min to resuspend the bacteria.

### Fluorescence measurement

Fluorescence was measured using an all-fibre spectroscopic system (optrode) [17]. The excitation source was a 473-nm solid-state laser placed behind shutter and controlled by a data acquisition (DAQ) card to prevent photobleaching the sample and ensure synchronisation with the spectrometer to allow for exact quantification of the fluorescence signal. To monitor power fluctuations, a 2 × 2 fibre coupler was used to deliver half the excitation light to a photodiode and the signal collected by the DAQ card. The other half was used to illuminate the sample. A single fibre probe was used for excitation and fluorescence collection. All the fibres in the instrument were multimode low OH silica fibre (diameter 200 μm, NA 0.22; Thorlabs Inc., Newton, NJ, USA). A 495-nm long-pass filter before the spectrometer removed the excitation line. Spectra were collected from 400 to 790 nm using an Ocean Optics QE65000 spectrometer. The spectrometer had no slit; the fibre acts as a slit to give an effective slit width of 200 μm. *E. coli* did not present any native fluorescence using 473 nm excitation.

Each measurement was acquired using a laser power of approximately 10 mW at the sample with 8 ms integration time. The sample was mixed using a vortex before positioning the tip of the sample fibre probe in the middle of the sample and taking a measurement. The fibre probe was washed with 70% ethanol between each measurement. Regularly, a blank measurement was made to ensure the fibre probe was clean.

### Data analysis

Dark noise was subtracted from each measurement. Each spectrum was normalised to a laser power of 10 mW and integration time of 8 ms. Subsequently, the background spectrum (saline) was subtracted from sample spectra. Any spectra with saturated signals were noted and were not included in a data matrix containing normalised spectra. All data analyses were executed using MATLAB R2014b version 8.4.0.150421 (The MathWorks, Natick, USA) software.

### Independent component analysis

ICA was applied to the fluorescence spectra collected from AO-dyed bacterial solutions. If a sample is composed of multiple fluorophores, then each of these fluorophores will contribute to the fluorescence spectrum. Each fluorophore will have a specific fluorescence signature when using the excitation wavelength of interest. The amount this fluorophore contributes to the fluorescence spectrum from the sample will depend on concentration of fluorophore within the solution. Hence, a sample fluorescence spectrum is the sum of the constituent fluorophores scaled to their respective concentrations. ICA can be used to identify the spectra of the fluorophores within a sample and their proportions in the analysed sample [18].

ICA aims to maximise the statistical independence and non-Gaussianity of the pure underlying signals, which are called the independent components (ICs). There are different methods to define and maximise statistical independence. In this study, we have chosen the joint approximate diagonalization of Eigen matrices (JADE) algorithm [19]. The JADE algorithm was used because it avoids convergence issues encountered with other algorithms (e.g. FastICA) [18]. The Matlab codes of this algorithm were downloaded from http://perso.telecom-paristech.fr/%7Ecardoso/Algo/Jade/jadeR.m. ICA-by-blocks was used to determine how many ICs should be extracted for the data set [20]. In this instance, the calibration spectral dataset was divided into two blocks; data from individual days were collected into the same block. Several ICA models were calculated for each block, with one to five ICs. The models calculated for each block for a given number of ICs were compared. ICs representative of true fluorophore signals should be found in each representative subset of the overall data matrix, and these ICs from the respective blocks will be strongly correlated. If too many ICs are extracted, the ICs will contain noise characteristic of the block, and hence, these ICs from the respective blocks will not be highly correlated. Once the appropriate number of ICs was determined, ICA was applied to the full calibration dataset and the IC weightings for each sample were subsequently used in classification models for bacterial concentration.

The Matlab function *mahal* was used to calculate the Mahalanobis distances of each data point to clusters of data for each order of magnitude of bacterial concentration. Mahalanobis distance measures the distance between a point and a distribution, i.e. it determines how many standard deviations a sample is away from mean of a group [21]. The Mahalanobis distance calculated, which follows a chi-square distribution, was used to determine significance, *p* < 0.01 (2 degrees of freedom). The limit of detection was the lowest bacterial concentration that was significantly different from all other orders of magnitude investigated.

### Bacterial concentration classification model

The IC weights of each sample were used to calculate classification models for the order of magnitude of the bacterial concentration. For each staining protocol, the corresponding calibration data was clustered by magnitude of bacterial concentration and the mean for each cluster was calculated. Using IC signals, the projection matrix of the validation data set was calculated. Each validation sample measurement was then assigned to an order of magnitude of bacterial concentration based on IC weights. This was done by calculating the squared Euclidean distance from the validation sample to the mean of each cluster. The validation sample was assigned to the closest cluster. Correct classification rates are the percentage of validation samples correctly assigned to magnitude of bacterial concentration.

## Results

### The AO fluorescence spectra

**Fig. 1:**
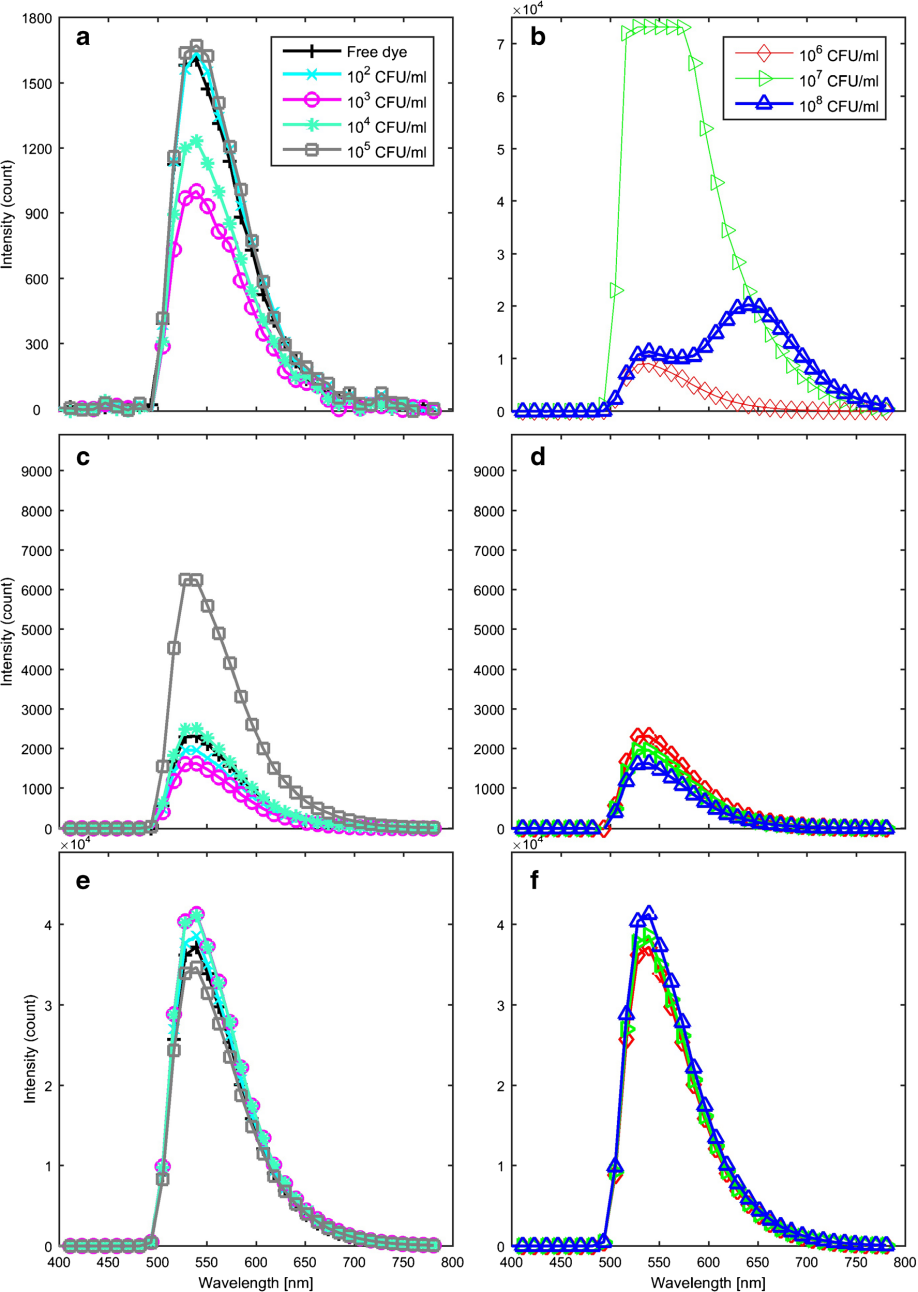
Exemplar spectra collected for (**a**) and (**b**) 2 × 10^−2^% AO staining followed by 3 washing cycles (**c**) and (**d**) 2 × 10^−3^% AO staining followed by two washing cycles and (**e**) and (**f**) 2 × 10^−4^% AO staining for 0–10^5^ CFU ml^−1^ *E. coli* in (**a**, **c** and **e**), and for 10^6^−10^8^ CFU ml^−1^ *E. coli* in (**b**, **d** and **f**). Spectra are coloured by concentration: *blue* 10^8^, *green* 10^7^, *red* 10^6^, *grey* 10^5^, *mint green* 10^4^, *magenta* 10^3^, *cyan* 10^2^, *black* 0 CFU ml^−1^

The fluorescence spectra of AO bound to bacteria is dominated by two peaks at approximately 535 and 650 nm. The position and intensity of these peaks shifted with bacterial concentration. Figure 1 represents exemplar spectra for each staining protocol, as indicated in the caption. Solutions containing 10^6^ CFU ml^−1^ *E. coli* or less (Fig. 1a) had a single peak at ∼535 nm which shifted to shorter wavelengths at lower concentrations of bacteria. The second peak at ∼650 nm was present for higher bacterial concentrations and shifted to shorter wavelengths to become a shoulder (∼625 nm) of the first peak at lower bacterial concentrations (Fig. 1b, c). Fluorescence signals for all 10^7^ CFU ml^−1^ *E. coli* samples (*N* = 7) stained with 2 × 10^−2^% AO followed by three washing cycles were saturated and therefore excluded from the ICA.

**Fig. 2:**
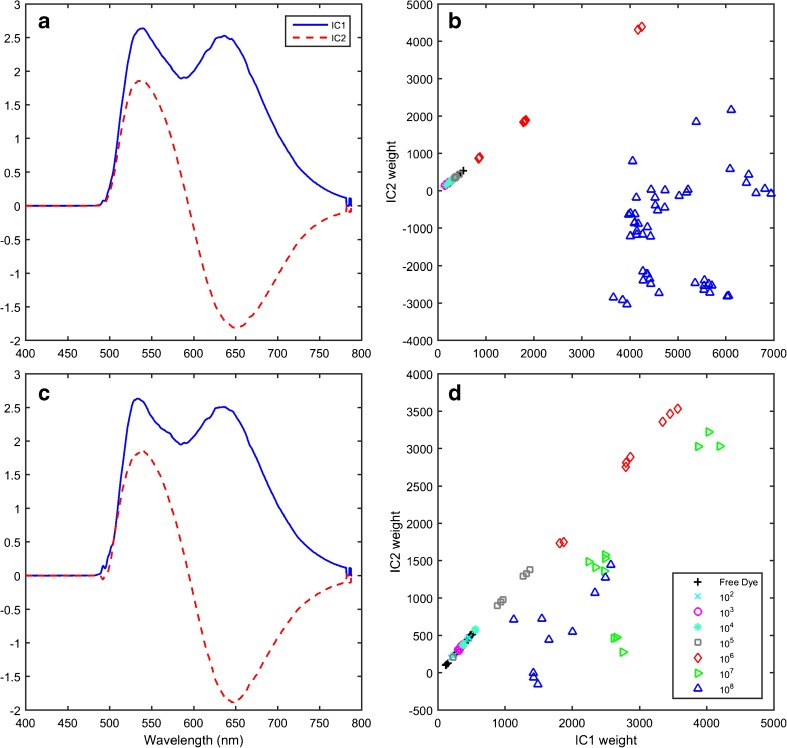
Independent component (IC) 1 (*solid*) and IC2 (*dashed*) signals (**a**) and weights (**b**) obtained from the 2 × 10^−2^% AO stain followed by 3 washing cycles data set (*N* = 39, *n* = 113). Note that the 10^7^ CFU ml^−1^ samples are absent from this independent component analysis since the signal was saturated. IC1 (*solid*) and IC2 (*dashed*) signals (**c**) and weights (**d**) obtained from the 2 × 10^−3^% AO stain followed by 2 washing cycles data set (*N* = 32, *n* = 76). Samples in (**c**) and (**d**) are coloured by concentration following the colour scheme of Fig. 1 and use the following symbols: *upward-pointing triangle* 10^8^, *left-pointing triangle* 10^7^, *diamond* 10^6^, *square* 10^5^, *asterisk* 10^4^, *circle* 10^3^, *cross* 10^2^, *plus sign* 0 CFU ml^−1^.

**Fig. 3:**
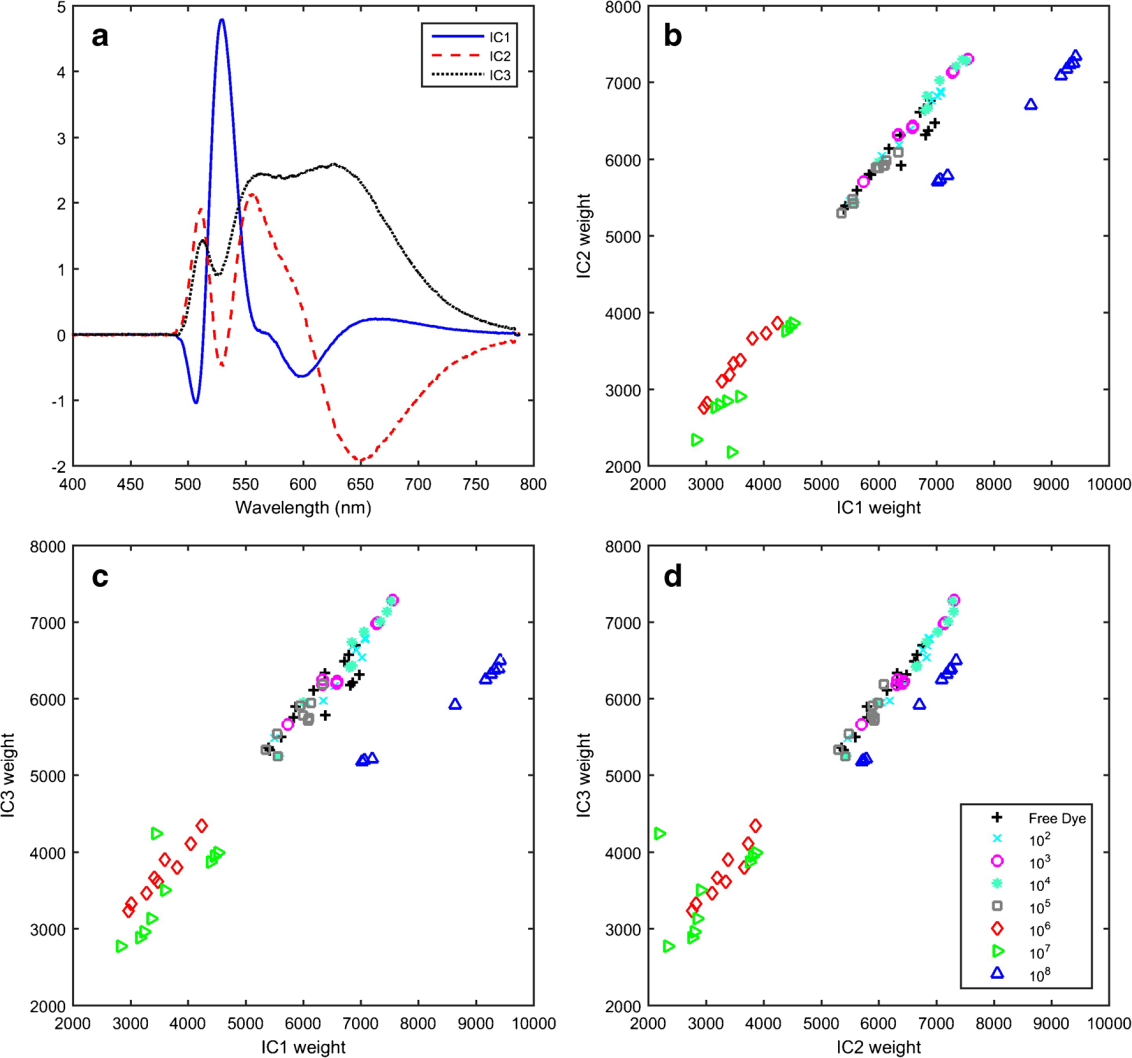
Independent component (IC) 1 (*solid*), IC2 (*dashed*) and IC3 (*dotted*) signals (**a**) and weights (**b**–**d**) obtained from the 2 × 10^−4^% AO staining procedure (*N* = 26, *n* = 76). Samples in (**b**), (**c**) and (**d**) are coloured by concentration following the colour scheme of Fig. 1 and use the following symbols: *upward-pointing triangle* 10^8^, *left-pointing triangle* 10^7^, *diamond* 10^6^, *square* 10^5^, *asterisk* 10^4^, *circle* 10^3^, *cross* 10^2^, *plus sign* 0 CFU ml^−1^

ICA was applied to data from each sample treatment individually. For the treatments 2 × 10^−2^% AO followed by three washing cycles and 2 × 10^−3^% AO followed by two washing cycles, two ICs described each spectral dataset (Fig. 2). The two ICs calculated were similar for each dataset and were dominated by features coincident with the two major peaks of AO bound in bacteria. IC1 loading had two peaks of similar intensity at 535 and 650 nm, and IC2 was the difference between these two peaks. For the sample treatment protocol 2 × 10^−4^% AO with no washing, three ICs were needed (Fig. 3). The third IC was dominated by an intense peak at ∼535 nm.

Across all three staining treatments, fluorescence spectra measured from samples containing 10^6^–10^8^ CFU ml^−1^ *E. coli* were significantly different (*p* < 0.01 based on Mahalanobis distance test) to fluorescence spectra measured from solutions containing free dye and <10^6^ CFU ml^−1^ *E. coli*. Furthermore, fluorescence spectra measured from all samples containing 10^5^ CFU ml^−1^ *E. coli* and stained with 2 × 10^−3^% AO followed by two washing cycles were significantly different (*p* < 0.01) from other bacterial concentrations.

### Classification models

Classification models for each staining treatment were calculated and validated with an external sample set (Table 1 and Electronic Supplementary Material (ESM) Tables S1–S4). Using the staining treatment 2 × 10^−2^% AO with three washing cycles, 100% of the validation samples were correctly classified. The correct order for magnitude was predicted for 77% of validation samples when using the staining conditions 2 × 10^−3^% AO followed by two washing cycles. The classification performance using this staining procedure was improved to 84% by adding a class for 10^5^ CFU ml^−1^ (Table 2). The 2 × 10^−4^% AO staining protocol had the lowest agreement rate at 69%.

**Table 1:** The agreement between fluorescence spectroscopy classification models and plate count in determining the order of magnitude of *E. coli* concentrations (<10^6^, 10^6^, 10^7^ or 10^8^ CFU ml^−1^) in suspension for the *N* = 13 validation samples. For classification, the first two independent component (IC) weights of validation samples treated with 2 × 10^−2^% AO followed three washing cycles and 2 × 10^−3^% AO followed by two washing cycles were used and the first three IC weights for validation samples treated with 2 × 10^−4^% AO

Plate count (order of CFU ml^−1^)	No. of samples	Staining procedure		
		2 × 10^−4^% AO, no washing (%)	2 × 10^−3^% AO, 2 washing cycles (%)	2 × 10^−2^% AO, 3 washing cycles (%)
<10^6^	4	75	75	100
10^6^	4	75	100	100
10^7^	3	33	33	100
10^8^	2	100	100	100

**Table 2:** The agreement between fluorescence spectroscopy classification models and plate count in determining the order of magnitude of *E. coli* concentrations (<10^5^, 10^5^ from the Table 1 <10^6^ data, *N* = 4) in solution when using 2 × 10^−3^% AO followed by two washing cycles. For classification, the first two independent component weights of validation samples were used

Plate count (order of CFU ml^−1^)	No. of samples	2 × 10^−3^% AO, 2 washing cycles (%)
<10^5^	2	100
10^5^	2	100

## Discussion

Several staining protocols were trialled in this study. The outcome of AO staining is influenced by a number of variables including the ratio of AO to nucleic acid [22]. In this study, a large bacterial concentration range was investigated (10^2^–10^8^ CFU ml^−1^); hence, several staining concentrations of AO were investigated. In addition to the concentration of AO, the number of washing steps was altered to eliminate the influence of unbound AO on the fluorescence spectra measured.

The intensity of AO fluorescence was not linear with bacterial concentration, in agreement with previous observations that the ratio of AO dye to nucleic acid affects the fluorescence signal. However, unique fluorescence profiles were observed for several AO dye-bacterial concentrations pairs, and this information was used to calculate classification models for the order of magnitude of bacterial concentration.

ICA was applied to each sample treatment data set individually. ICs with negative peaks were observed for all data sets. The aim of ICA here was to calculate the spectra of the underlying fluorophores and the respective weighting of each in a sample spectrum. If two components are inter-dependent (i.e. fluorophore 1 increases as fluorophore 2 decreases), these signals are not independent [18]. Consequently, one IC will have contributions from both these components; the IC will not represent a ‘pure’ component but rather a single phenomenon. The peak centre around 650 nm increased in intensity as the peak centred around 535 nm decreased in intensity, and this behaviour is illustrated in calculated ICs for each dataset. As bacterial concentration increases, the width of the 535 nm peak increases and develops a higher wavelength shoulder and, subsequently, a peak at 650 nm when a high-enough concentration of AO is used to stain the sample. When using 2 × 10^−3^% AO followed by two washing cycles to prepare samples, the peak at 650 nm was not obvious in the collected fluorescence spectra; however, the ICA indicates that these spectral changes were occurring within the dataset as both peaks are present within calculated ICs. Three ICs were appropriate to describe the dataset collected from samples prepared with 2 × 10^−4^% AO. One of these ICs was dominated by a sharp peak at 535 nm, which probably represents the contribution of unbound AO to the collected fluorescence spectra. The other two ICs were similar to the ICs calculated for the other staining treatments, with the exception of a minimum at 535 nm. This minimum is likely a result of interference from unbound AO and potentially indicates that the 2 × 10^−4^% AO dataset is not as well described by the independent components analysis as the 2 × 10^−2^% AO followed by three washing cycles and 2 × 10^−3^% AO followed by two washing cycles datasets.

The staining protocol that classifies the most samples correctly was 2 × 10^−2^% AO stain followed by three washing cycles. All validation samples down to 10^5^ CFU ml^−1^ were correctly classified using this staining treatment. The ∼535 nm peak intensities collected from 10^6^ CFU ml^−1^ solutions were at least twice as large as those from solutions containing less than 10^6^ CFU ml^−1^. The saturation of signals from 10^7^ CFU ml^−1^ solutions (not included in the ICA and plots) provided a clear separation from the single peak spectra of 10^6^ CFU ml^−1^ solutions and from the spectra with a shoulder or two peaks collected from 10^8^ CFU ml^−1^ solutions. This saturation was a quick indicator of a bacterial concentration of 10^7^ CFU ml^−1^ without the need for further analysis.

Other staining treatments were investigated where the concentration of AO was reduced, as were the number of washing cycles. The 2 × 10^−3^% AO followed by two washing cycles staining treatment did not correctly classify two of the validation samples; however, this treatment did show promise with respect to the classification of 10^5^ CFU ml^−1^ samples. As such, this staining protocol is a good complement to further classify the low concentration sample identified by the previous protocol as below 10^6^ CFU ml^−1^. The 2 × 10^−4^% AO staining protocol had the lowest agreement rate, failing to identify solutions in the order of 10^7^ CFU ml^−1^. The fluorescence of 10^6^ and 10^7^ CFU ml^−1^ samples was quenched relative to the fluorescence signal of all other bacteria concentrations, and since the solutions were not washed, the fluorescence of the free dye in the solution concealed the fluorescence from bound AO. The difference in the fluorescence of 10^6^ and 10^7^ CFU ml^−1^ samples were noticed when samples were washed. The sample containing 6.8 × 10^7^ CFU ml^−1^ was predicted to be 10^8^ CFU ml^−1^, similar to the 2 × 10^−3^% AO and two washing cycles staining protocol.

**Fig. 4:**
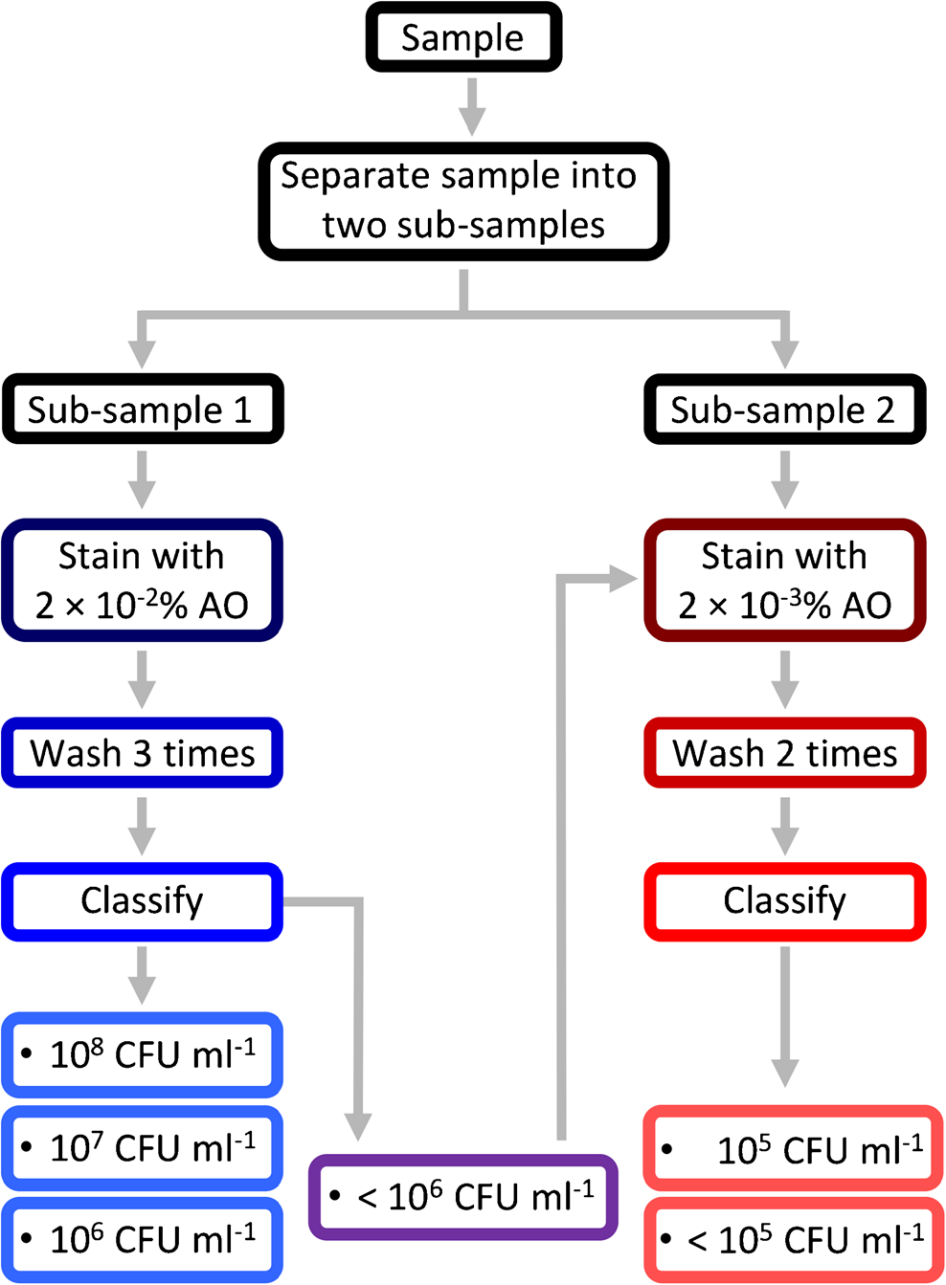
Flow chart of the final method to be used to quantify samples from 10^8^ CFU ml^−1^ down to 10^5^ CFU ml^−1^, or below 10^5^ CFU ml^−1^, using our device and technique

The major drawback in the method we describe is the strong signal from free AO when the concentration of bacteria is low compared to the concentration of dye. Our method (Fig. 4) therefore requires several washing steps that may lead to precision and/or accuracy issues. To overcome this limitation, we are currently investigating (a) other dyes that have a higher quantum yield when bound to nucleic acid [23] and (b) automating the staining/washing protocol by using microfluidic devices [24, 25]. We believe that both of the above should contribute to the development of a device with a sensitivity relevant for industrial applications. To date, the device described in this study has been developed to offer a competitive alternative to plate counts in terms of time and costs. The actual costs of our technique relate to initial setup costs for the device and ongoing reagent costs. At present, a single prototype device can be constructed for US$20,000, but larger scale production and replacement of expensive elements may substantially reduce this. We anticipate consumable tests will be low cost; at present, these are around US$10 per test, which is competitive with agar plate testing, but substantially faster (20 min compared to days) and requiring less technician time.

Overall, we conclude this study showed that our cost-effective fluorescence spectroscopy method can be used to determine the order of magnitude of bacterial concentrations greater than 10^5^ CFU ml^−1^ using the procedure, as illustrated in Fig. 4. Staining with 2 × 10^−2^% AO stain followed by three washing cycles can be used to determine whether a solution contains <10^6^, 10^6^, 10^7^ or 10^8^ CFU ml^−1^. Samples with less than 10^6^ CFU ml^−1^ can be tested using the staining protocol 2 × 10^−3^% AO stain followed by two washing cycles to determine whether the sample has a bacterial concentration in the order of 10^5^ CFU ml^−1^ or less than 10^5^ CFU ml^−1^. We expect that increasing the amount of calibration data will improve the classification accuracy and enable the better prediction of bacterial content of a sample. In addition, future studies should incorporate multiple species of bacteria to calculate a general model for the enumeration of bacteria by order of magnitude. We also propose to validate the protocol developed on real samples in the laboratory and then in the field in collaboration with the food industry.

The method developed in this study is fast in comparison with traditional counting methods such as AODC, where it has been reported that it can take a well-trained individual approximately 1 h per sample (using triplicate filters) to perform a count [26]. Other prototype small-scale spectrophotometers are described in the literature; however, these examples have been applied to either measuring bacterial growth by turbidity [27] or identifying the load of specific organisms by quantifying enzyme activities with fluorogenic substrates [28]. In both cases, the limit of detection and time to detection do not compare favourably with the combination of stain and spectrophotometer we describe here. Our method requires minimal operator expertise; measurement of the sample requires simply immersing a probe into the solution. The longest form of sample preparation trialled here required 25 min, and measurements required less than a minute. Fast methods for the detection of bacterial concentration by order of magnitude could be useful in biocide testing or other areas of microbiology where the Miles-Misra plating technique [29] is typically employed.

## Electronic supplementary material

ESM 1 (PDF 119 kb)
