# Supplementary material for: A rapid and low-cost estimation of bacteria counts in solution using fluorescence spectroscopy
Source: Anal Bioanal Chem. 2017 Apr 7;409(16):3959–67. doi: 10.1007/s00216-017-0347-1 (PMC5437196; doi:10.1007/s00216-017-0347-1)
Supplement: Supplementary file 1 — (PDF 119 kb) [file 216_2017_347_MOESM1_ESM.pdf]

Analytical and Bioanalytical Chemistry

Electronic Supplementary Material

A rapid and low-cost estimation of bacteria counts in solution using fluorescence spectroscopy

Rachel Guo, Cushla McGoverin, Simon Swift, Frederique Vanholsbeeck

Table S1 Test set validation classification results for the 2 x 10^{-2} % AO stain followed by 3 washing cycles data set. This classification was based on the minimum squared Euclidean distance of independent component weights 1 and 2. 10^7 CFU ml^{-1} samples were excluded from this analysis as the signal was saturated. Sample groupings were $\leq 10^5$ CFU ml^{-1}, 10^6 CFU ml^{-1}, 10^7 CFU ml^{-1}, and 10^8 CFU ml^{-1}

Sample	Actual	Classified	Agreement
1a	$\leq 10^5$ CFU ml^{-1}	$\leq 10^5$ CFU ml^{-1}	Yes
1b	$\leq 10^5$ CFU ml^{-1}	$\leq 10^5$ CFU ml^{-1}	Yes
1c	$\leq 10^5$ CFU ml^{-1}	$\leq 10^5$ CFU ml^{-1}	Yes
2a	$\leq 10^5$ CFU ml^{-1}	$\leq 10^5$ CFU ml^{-1}	Yes
2b	$\leq 10^5$ CFU ml^{-1}	$\leq 10^5$ CFU ml^{-1}	Yes
2c	$\leq 10^5$ CFU ml^{-1}	$\leq 10^5$ CFU ml^{-1}	Yes
3a	$\leq 10^5$ CFU ml^{-1}	$\leq 10^5$ CFU ml^{-1}	Yes
3b	$\leq 10^5$ CFU ml^{-1}	$\leq 10^5$ CFU ml^{-1}	Yes
3c	$\leq 10^5$ CFU ml^{-1}	$\leq 10^5$ CFU ml^{-1}	Yes
4a	$\leq 10^5$ CFU ml^{-1}	$\leq 10^5$ CFU ml^{-1}	Yes
4b	$\leq 10^5$ CFU ml^{-1}	$\leq 10^5$ CFU ml^{-1}	Yes
4c	$\leq 10^5$ CFU ml^{-1}	$\leq 10^5$ CFU ml^{-1}	Yes
5a	10^6 CFU ml^{-1}	10^6 CFU ml^{-1}	Yes
5b	10^6 CFU ml^{-1}	10^6 CFU ml^{-1}	Yes
5c	10^6 CFU ml^{-1}	10^6 CFU ml^{-1}	Yes
6a	10^6 CFU ml^{-1}	10^6 CFU ml^{-1}	Yes
6b	10^6 CFU ml^{-1}	10^6 CFU ml^{-1}	Yes
6c	10^6 CFU ml^{-1}	10^6 CFU ml^{-1}	Yes
7a	10^6 CFU ml^{-1}	10^6 CFU ml^{-1}	Yes
7b	10^6 CFU ml^{-1}	10^6 CFU ml^{-1}	Yes
8a	10^6 CFU ml^{-1}	10^6 CFU ml^{-1}	Yes
8b	10^6 CFU ml^{-1}	10^6 CFU ml^{-1}	Yes
8c	10^6 CFU ml^{-1}	10^6 CFU ml^{-1}	Yes
12a	10^8 CFU ml^{-1}	10^8 CFU ml^{-1}	Yes
12b	10^8 CFU ml^{-1}	10^8 CFU ml^{-1}	Yes
12c	10^8 CFU ml^{-1}	10^8 CFU ml^{-1}	Yes
13a	10^8 CFU ml^{-1}	10^8 CFU ml^{-1}	Yes
13b	10^8 CFU ml^{-1}	10^8 CFU ml^{-1}	Yes
13c	10^8 CFU ml^{-1}	10^8 CFU ml^{-1}	Yes

Table S2 Test set validation classification results for the 2 x 10^{-3} % AO stain followed by 3 washing cycles data set. This classification was based on the minimum squared Euclidean distance of independent component weights 1 and 2. Sample groupings were $\leq 10^5$ CFU ml^{-1}, 10^6 CFU ml^{-1}, 10^7 CFU ml^{-1}, and 10^8 CFU ml^{-1}

Sample	Actual	Classified	Agreement
1a	$\leq 10^5$ CFU ml^{-1}	$\leq 10^5$ CFU ml^{-1}	Yes
1b	$\leq 10^5$ CFU ml^{-1}	$\leq 10^5$ CFU ml^{-1}	Yes
1c	$\leq 10^5$ CFU ml^{-1}	$\leq 10^5$ CFU ml^{-1}	Yes
2a	$\leq 10^5$ CFU ml^{-1}	$\leq 10^5$ CFU ml^{-1}	Yes
2b	$\leq 10^5$ CFU ml^{-1}	$\leq 10^5$ CFU ml^{-1}	Yes
2c	$\leq 10^5$ CFU ml^{-1}	$\leq 10^5$ CFU ml^{-1}	Yes
3a	$\leq 10^5$ CFU ml^{-1}	$\leq 10^5$ CFU ml^{-1}	Yes
3b	$\leq 10^5$ CFU ml^{-1}	$\leq 10^5$ CFU ml^{-1}	Yes
3c	$\leq 10^5$ CFU ml^{-1}	$\leq 10^5$ CFU ml^{-1}	Yes
4a	$\leq 10^5$ CFU ml^{-1}	10^8 CFU ml^{-1}	No
4b	$\leq 10^5$ CFU ml^{-1}	10^8 CFU ml^{-1}	No
4c	$\leq 10^5$ CFU ml^{-1}	10^8 CFU ml^{-1}	No
5a	10^6 CFU ml^{-1}	10^6 CFU ml^{-1}	Yes
5b	10^6 CFU ml^{-1}	10^6 CFU ml^{-1}	Yes
5c	10^6 CFU ml^{-1}	10^6 CFU ml^{-1}	Yes
6a	10^6 CFU ml^{-1}	10^6 CFU ml^{-1}	Yes
6b	10^6 CFU ml^{-1}	10^6 CFU ml^{-1}	Yes
6c	10^6 CFU ml^{-1}	10^6 CFU ml^{-1}	Yes
7a	10^6 CFU ml^{-1}	10^6 CFU ml^{-1}	Yes
7b	10^6 CFU ml^{-1}	10^6 CFU ml^{-1}	Yes
7c	10^6 CFU ml^{-1}	10^6 CFU ml^{-1}	Yes
8a	10^6 CFU ml^{-1}	10^6 CFU ml^{-1}	Yes
8b	10^6 CFU ml^{-1}	10^6 CFU ml^{-1}	Yes
8c	10^6 CFU ml^{-1}	10^6 CFU ml^{-1}	Yes
9a	10^7 CFU ml^{-1}	10^6 CFU ml^{-1}	No
9b	10^7 CFU ml^{-1}	10^6 CFU ml^{-1}	No
9c	10^7 CFU ml^{-1}	10^6 CFU ml^{-1}	No
10a	10^7 CFU ml^{-1}	10^7 CFU ml^{-1}	Yes
10b	10^7 CFU ml^{-1}	10^7 CFU ml^{-1}	Yes
10c	10^7 CFU ml^{-1}	10^7 CFU ml^{-1}	Yes
11a	10^7 CFU ml^{-1}	10^8 CFU ml^{-1}	No
11b	10^7 CFU ml^{-1}	10^8 CFU ml^{-1}	No
11c	10^7 CFU ml^{-1}	10^8 CFU ml^{-1}	No
12a	10^8 CFU ml^{-1}	10^8 CFU ml^{-1}	Yes
12b	10^8 CFU ml^{-1}	10^8 CFU ml^{-1}	Yes
12c	10^8 CFU ml^{-1}	10^8 CFU ml^{-1}	Yes
13a	10^8 CFU ml^{-1}	10^8 CFU ml^{-1}	Yes
13b	10^8 CFU ml^{-1}	10^8 CFU ml^{-1}	Yes
13c	10^8 CFU ml^{-1}	10^8 CFU ml^{-1}	Yes

Table S3 Test set validation classification results for the 2 x 10^{-3} % AO stain followed by 3 washing cycles data set. This classification was based on the minimum squared Euclidean distance of independent component weights 1 and 2. Sample groupings were ≤10^4 CFU ml^{-1}, 10^5 CFU ml^{-1}, 10^6 CFU ml^{-1}, 10^7 CFU ml^{-1}, and 10^8 CFU ml^{-1}

Sample	Actual	Classified	Agreement
1a	≤10^4 CFU ml^{-1}	≤10^4 CFU ml^{-1}	Yes
1b	≤10^4 CFU ml^{-1}	≤10^4 CFU ml^{-1}	Yes
1c	≤10^4 CFU ml^{-1}	≤10^4 CFU ml^{-1}	Yes
2a	≤10^4 CFU ml^{-1}	≤10^4 CFU ml^{-1}	Yes
2b	≤10^4 CFU ml^{-1}	≤10^4 CFU ml^{-1}	Yes
2c	≤10^4 CFU ml^{-1}	≤10^4 CFU ml^{-1}	Yes
3a	10^5 CFU ml^{-1}	10^5 CFU ml^{-1}	Yes
3b	10^5 CFU ml^{-1}	10^5 CFU ml^{-1}	Yes
3c	10^5 CFU ml^{-1}	10^5 CFU ml^{-1}	Yes
4a	10^5 CFU ml^{-1}	10^5 CFU ml^{-1}	Yes
4b	10^5 CFU ml^{-1}	10^5 CFU ml^{-1}	Yes
4c	10^5 CFU ml^{-1}	10^5 CFU ml^{-1}	Yes
5a	10^6 CFU ml^{-1}	10^6 CFU ml^{-1}	Yes
5b	10^6 CFU ml^{-1}	10^6 CFU ml^{-1}	Yes
5c	10^6 CFU ml^{-1}	10^6 CFU ml^{-1}	Yes
6a	10^6 CFU ml^{-1}	10^6 CFU ml^{-1}	Yes
6b	10^6 CFU ml^{-1}	10^6 CFU ml^{-1}	Yes
6c	10^6 CFU ml^{-1}	10^6 CFU ml^{-1}	Yes
7a	10^6 CFU ml^{-1}	10^6 CFU ml^{-1}	Yes
7b	10^6 CFU ml^{-1}	10^6 CFU ml^{-1}	Yes
7c	10^6 CFU ml^{-1}	10^6 CFU ml^{-1}	Yes
8a	10^6 CFU ml^{-1}	10^6 CFU ml^{-1}	Yes
8b	10^6 CFU ml^{-1}	10^6 CFU ml^{-1}	Yes
8c	10^6 CFU ml^{-1}	10^6 CFU ml^{-1}	Yes
9a	10^7 CFU ml^{-1}	10^6 CFU ml^{-1}	No
9b	10^7 CFU ml^{-1}	10^6 CFU ml^{-1}	No
9c	10^7 CFU ml^{-1}	10^6 CFU ml^{-1}	No
10a	10^7 CFU ml^{-1}	10^7 CFU ml^{-1}	Yes
10b	10^7 CFU ml^{-1}	10^7 CFU ml^{-1}	Yes
10c	10^7 CFU ml^{-1}	10^7 CFU ml^{-1}	Yes
11a	10^7 CFU ml^{-1}	10^8 CFU ml^{-1}	No
11b	10^7 CFU ml^{-1}	10^8 CFU ml^{-1}	No
11c	10^7 CFU ml^{-1}	10^8 CFU ml^{-1}	No
12a	10^8 CFU ml^{-1}	10^8 CFU ml^{-1}	Yes
12b	10^8 CFU ml^{-1}	10^8 CFU ml^{-1}	Yes
12c	10^8 CFU ml^{-1}	10^8 CFU ml^{-1}	Yes
13a	10^8 CFU ml^{-1}	10^8 CFU ml^{-1}	Yes
13b	10^8 CFU ml^{-1}	10^8 CFU ml^{-1}	Yes
13c	10^8 CFU ml^{-1}	10^8 CFU ml^{-1}	Yes

Table S4 Test set validation classification results for the 2 x 10^{-4} % AO stain followed by 3 washing cycles data set. This classification was based on the minimum squared Euclidean distance of independent component weights 1 and 2. Sample groupings were ≤10^5 CFU ml^{-1}, 10^6 CFU ml^{-1}, 10^7 CFU ml^{-1}, and 10^8 CFU ml^{-1}

Sample	Actual	Classified	Agreement
1a	≤10^5 CFU ml^{-1}	≤10^8 CFU ml^{-1}	No
1b	≤10^5 CFU ml^{-1}	≤10^8 CFU ml^{-1}	No
1c	≤10^5 CFU ml^{-1}	≤10^8 CFU ml^{-1}	No
2a	≤10^5 CFU ml^{-1}	≤10^5 CFU ml^{-1}	Yes
2b	≤10^5 CFU ml^{-1}	≤10^5 CFU ml^{-1}	Yes
2c	≤10^5 CFU ml^{-1}	≤10^5 CFU ml^{-1}	Yes
3a	≤10^5 CFU ml^{-1}	≤10^5 CFU ml^{-1}	Yes
3b	≤10^5 CFU ml^{-1}	≤10^5 CFU ml^{-1}	Yes
3c	≤10^5 CFU ml^{-1}	≤10^5 CFU ml^{-1}	Yes
4a	≤10^5 CFU ml^{-1}	10^5 CFU ml^{-1}	Yes
4b	≤10^5 CFU ml^{-1}	10^5 CFU ml^{-1}	Yes
4c	≤10^5 CFU ml^{-1}	10^5 CFU ml^{-1}	Yes
5a	10^6 CFU ml^{-1}	10^6 CFU ml^{-1}	Yes
5b	10^6 CFU ml^{-1}	10^6 CFU ml^{-1}	Yes
5c	10^6 CFU ml^{-1}	10^6 CFU ml^{-1}	Yes
6a	10^6 CFU ml^{-1}	10^6 CFU ml^{-1}	Yes
6b	10^6 CFU ml^{-1}	10^6 CFU ml^{-1}	Yes
6c	10^6 CFU ml^{-1}	10^6 CFU ml^{-1}	Yes
7a	10^6 CFU ml^{-1}	10^6 CFU ml^{-1}	Yes
7b	10^6 CFU ml^{-1}	10^6 CFU ml^{-1}	Yes
7c	10^6 CFU ml^{-1}	10^6 CFU ml^{-1}	Yes
8a	10^6 CFU ml^{-1}	10^7 CFU ml^{-1}	No
8b	10^6 CFU ml^{-1}	10^7 CFU ml^{-1}	No
8c	10^6 CFU ml^{-1}	10^7 CFU ml^{-1}	No
9a	10^7 CFU ml^{-1}	10^7 CFU ml^{-1}	Yes
9b	10^7 CFU ml^{-1}	10^7 CFU ml^{-1}	Yes
9c	10^7 CFU ml^{-1}	10^7 CFU ml^{-1}	Yes
10a	10^7 CFU ml^{-1}	10^6 CFU ml^{-1}	No
10b	10^7 CFU ml^{-1}	10^6 CFU ml^{-1}	No
10c	10^7 CFU ml^{-1}	10^6 CFU ml^{-1}	No
11a	10^7 CFU ml^{-1}	10^8 CFU ml^{-1}	No
11b	10^7 CFU ml^{-1}	10^8 CFU ml^{-1}	No
11c	10^7 CFU ml^{-1}	10^8 CFU ml^{-1}	No
12a	10^8 CFU ml^{-1}	10^8 CFU ml^{-1}	Yes
12b	10^8 CFU ml^{-1}	10^8 CFU ml^{-1}	Yes
12c	10^8 CFU ml^{-1}	10^8 CFU ml^{-1}	Yes
13a	10^8 CFU ml^{-1}	10^8 CFU ml^{-1}	Yes
13b	10^8 CFU ml^{-1}	10^8 CFU ml^{-1}	Yes
13c	10^8 CFU ml^{-1}	10^8 CFU ml^{-1}	Yes
